# Improving polygenic prediction from whole-genome sequencing data by leveraging predicted epigenomic features

**DOI:** 10.1073/pnas.2419202122

**Published:** 2025-06-12

**Authors:** Wanwen Zeng, Hanmin Guo, Qiao Liu, Wing Hung Wong

**Affiliations:** ^a^Department of Statistics, Stanford University, Stanford, CA 94305; ^b^Bio-X Program, Stanford University, Stanford, CA 94305; ^c^Department of Psychiatry and Behavioral Sciences, Stanford University, Stanford, CA 94305; ^d^Department of Biomedical Data Science, Stanford University, Stanford, CA 94305

**Keywords:** genomic LLM, polygenic risk scores, epigenomics, UKBB

## Abstract

Polygenic risk scores (PRS) are essential tools for estimating individual susceptibility to complex diseases by aggregating the effects of many genetic variants. With the advent of whole-genome sequencing (WGS), rare and de novo variants can now be detected at scale, presenting new opportunities to enhance PRS performance. Additionally, regulatory mechanisms that govern gene expression play a critical role in disease manifestation, suggesting further potential for improvement. However, most existing PRS methods are not well-equipped to incorporate nonlinear variant effects, rare variant contributions, or regulatory context. To address these limitations, we developed Epi-PRS, a novel framework that leverages large language models (LLMs) to impute cell-type-specific epigenomic signals from personal diploid genotypes. These imputed signals act as informative intermediates between genotype and phenotype, allowing for more accurate modeling of variant impact. Our simulation studies demonstrate that Epi-PRS improves predictive accuracy by incorporating nonlinear relationships, rare variant effects, and regulatory information across large genomic regions. When applied to real data from the UK Biobank, Epi-PRS significantly outperforms existing PRS approaches in predicting risk for both breast cancer and type 2 diabetes. These results underscore the advantages of integrating WGS data, epigenomic context, and advanced LLMs framework to enhance both the predictive power and interpretability of PRS. Overall, Epi-PRS represents a promising step toward more precise and biologically informed disease risk prediction, with broad implications for advancing personalized medicine and understanding complex genetic architectures.

## Significance

Epi-PRS improves polygenic risk scoring by integrating genomic large language models (LLMs) to impute epigenomic signals as intermediaries between genotype and phenotype. This approach enables a more comprehensive assessment of personal variant impacts by incorporating nonlinear models, rare variants, and regulatory mechanisms. By leveraging the power of genomic LLM trained on massive amount of reference epigenomics data, Epi-PRS has demonstrated superior performance over existing PRS methods in predicting genetic risk for breast cancer and diabetes in UK Biobank data. These results highlight the potential of Epi-PRS to improve disease risk modeling and advance the field of precision medicine.

Polygenic risk score (PRS) has become pivotal in genetics for assessing an individual’s susceptibility to complex diseases (1–4). In personalized medicine, PRS enables stratification of individuals based on their genetic risk, facilitating targeted interventions and optimizing clinical outcomes (5, 6). This predictive capability is crucial for early disease detection and prevention, potentially reducing healthcare costs and improving personalized health care (7, 8). Furthermore, PRS elucidates the genetic architecture of complex traits, providing insights into the relevant biological pathways involved in disease etiology (9, 10), which may potentially guide the development of new therapeutic strategies and enhance our ability to identify potential drug targets (11, 12).

Detailed knowledge of how diversity in the human genome sequence affects phenotypic diversity relies on a comprehensive characterization of both sequences and phenotypic variations (13). Whole-genome sequencing (WGS) has advanced this field by enabling the detection of numerous rare and even de novo noncoding variants (14). WGS is more comprehensive than genotyping arrays and whole-exome sequencing (WES) as it allows for the detection of a broader range of variant types and provides more uniform coverage of the entire genome, including regulatory regions (15). Recently, very large-scale WGS data with associated clinical phenotypes are being made available for research. These include WGS data for about 500,000 participants from the UK Biobank^16^ and 150,000 participants from the US Million Veterans Program (16, 17). Clearly, there is an urgent need for PRS methods capable of utilizing the full information in WGS data.

Current methods for PRS prediction, such as LDpred2 (18, 19) and PRS-CS (20), have been instrumental in advancing our understanding of genetic risk and have provided invaluable insights into disease susceptibility. These methods were designed with the best available data at the time of their development, typically from SNP arrays that primarily capture common variants (1). As such, they have been highly effective within those contexts. However, with the advent of WGS and larger datasets, the landscape has evolved, revealing new opportunities for refinement. First, many PRS methods rely on linear models, which were well suited to the data of earlier studies, providing computational efficiency and ease of interpretation. However, linear models assume additive effects of genetic variants and may not fully capture the complex interactions between variants, even as the larger datasets offer rich opportunities to explore these interactions (21, 22). Thus, there is a need to develop nonlinear models to account for nonadditive effects in larger datasets (23). Second, given their focus on common variants, traditional PRS methods were not designed to incorporate rare and de novo variants. These rare variants, though less frequent, can have substantial impacts on disease risk (24), and incorporating them could enhance the predictive power of PRS (25, 26). Finally, the role of regulatory mechanisms in gene expression and disease manifestation has become increasingly clear (27–30). For example, genetic variants can influence disease risk not only through their direct effects on protein function but also by altering the activities of regulatory elements such as enhancers, promoters, and transcription factor binding sites (31–33). Current PRS methods either ignore this regulatory information (19, 20) or incorporate as linear model priors and parameters (34, 35). As our understanding of regulatory mechanisms expands, there are exciting opportunities to improve PRS models by integrating this critical layer of biological information (34, 36, 37).

To investigate the factors limiting the accuracy of current PRS methods, we conducted comprehensive simulation studies. First, we examined the limits imposed by linear models. Our simulations revealed that when diseases are influenced by complex interactions between genetic variants, nonlinear models can significantly outperform traditional linear models by capturing these intricate interactions and improving predictive performance. Second, we explored the role of rare variants. The simulation showed that current PRS methods are not effective in using the information from rare variants. Therefore, they performed poorly in predicting genetic risk for diseases where rare variants play a critical role.

To address these challenges, we developed Epi-PRS, a nonlinear method for polygenic prediction capable of using information from both common and rare variants. Our approach is to use the phased personal sequences to represent all variants in large genome regions, regardless of whether the variants are common, rare, or de novo. Specifically, for every individual in our study cohort, we use a genomic large language model (LLM) to transform the phased DNA sequences into a set of context-specific and region-specific epigenomic features. Then, we use these personal epigenomic features as explanatory variables to learn a nonlinear polygenic risk prediction model. This approach is motivated by the important role of regulatory mechanisms in disease etiology (27–30). For example, genetic variants can influence disease risk by altering the activities of regulatory elements such as enhancers, promoters, and transcription factor binding sites (31–33). With the advent of genomic LLMs that are learned from a massive amount of epigenomic datasets across diverse cellular contexts, we can now predict many types of epigenomic signals across a genomic region based on the DNA sequence of that region. By leveraging these LLMs, our approach offers a principled way to incorporate the effects of all variants via their impact on predicted epigenomic features. Moreover, the epigenomic features are biologically more meaningful as explanatory variables than the genotypes themselves. Thus, disease risk models based on predicted epigenomic features may offer deeper insight into the molecular basis of diseases. Our simulation studies demonstrated that this approach (Epi-PRS) significantly outperforms traditional PRS methods. Importantly, when applied to UK Biobank data on breast cancer and type 2 diabetes, Epi-PRS showed clear and substantial improvements over state-of-the-art PRS methods, highlighting its potential for enhancing disease risk prediction through the integration of advanced computational techniques and comprehensive genetic insights.

In summary, this study not only introduces a method for enhancing polygenic risk prediction but also provides insights into the factors affecting the performance of PRS methods. The simulation results suggest that existing PRS methods can be further strengthened by integrating nonlinear models, rare variants, and regulatory information, paving the way to significant advancements in disease risk modeling. Our findings provide useful perspectives on how to use WGS data to overcome the limitations of current PRS methods. It is hoped that our results can enable more precise genetic risk assessments and deeper understanding of the regulatory mechanisms underlying complex diseases.

## Results

### Overview of the Epi-PRS Model.

**Fig. 1.**
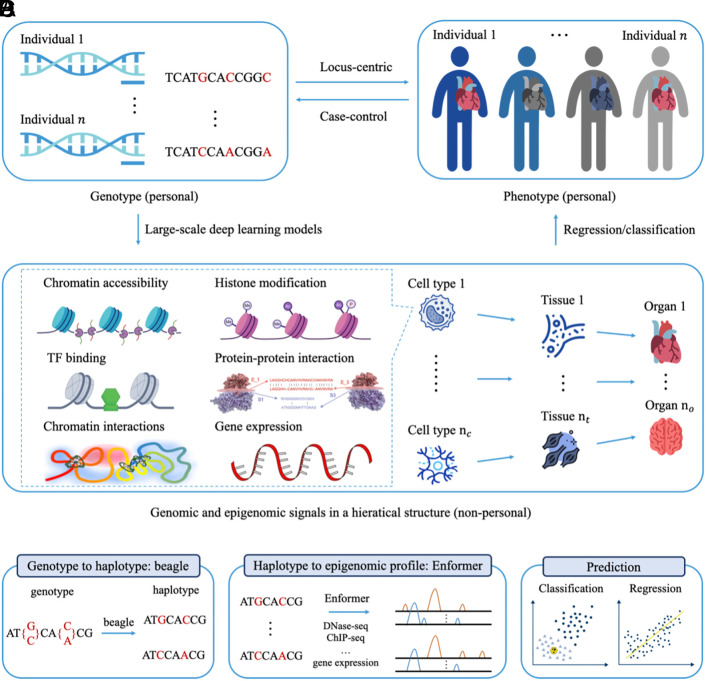
A simplified diagram that shows the understanding of how (*A*) personal genotypes affect (*B*) personal phenotypes, which requires (*C*) the modeling of the relationship between different layers of omics based on nonpersonal context-specific reference data using large-scale deep learning models. (*D*) Assuming WGS and phenotype data are available from the cohort, the main steps of Epi-PRS. 1) Personal genome construction: maternal and paternal genomes will be constructed from personal WGS as the input for Epi-PRS. 2) Epigenomic features extraction: genomic LLM (e.g., Enformer) will be applied to the haploid genome to obtain the personal epigenomic features. 3) Risk prediction: build model for disease risk as a function of the epigenomic features.

The Epi-PRS workflow introduces a comprehensive approach to disease risk prediction by integrating personal genomic and epigenomic data in unique ways. As shown in Fig. 1, Epi-PRS models how personal genotypes influence phenotypes through a diverse array of genomic and epigenomic profiles, imputed by a genomic LLM trained on reference data from a diverse range of cellular contexts. A distinguishing feature of Epi-PRS is its ability to handle diploid sequences, allowing for a more comprehensive assessment of variant interactions in personal DNA sequences, which enhances the accuracy of disease risk predictions. The Epi-PRS workflow comprises three major steps: personal genome construction, epigenomic feature extraction, and disease risk prediction. Detailed descriptions of the methods are provided in the *Methods* section.

Epi-PRS offers several unique aspects that extend beyond traditional PRS prediction approaches: 1) Integration of molecular phenotypes: While conventional methods focus primarily on genotype data, Epi-PRS incorporates imputed molecular phenotypes, providing additional context for how genetic variants may influence disease risk through regulatory mechanisms. 2) Diploid sequence modeling: Epi-PRS captures the combinatorial effects of multiple variants by processing diploid sequences, which is important for understanding the complexity of polygenic traits. 3) Inclusion of rare and de novo variants: Epi-PRS is able to include rare and de novo variants, expanding the scope of genetic information considered in disease risk prediction. These advancements build on the strong foundation laid by existing PRS methods and aim to provide additional perspectives for improving disease risk prediction.

### PRS Prediction on Simulation Data.

A series of simulation experiments were carefully designed and conducted to evaluate the performance of PRS methods. The evaluation focused on three key comparisons: 1) the efficacy of linear models versus nonlinear models; 2) incorporating regulatory information or not; and 3) considering the impact of rare variants or not. These comprehensive comparisons aimed to provide a deeper understanding of the factors influencing PRS accuracy and effectiveness. A regulatory-based phenotype simulation tool (RegPheno) was developed to ensure that the simulated phenotypes are affected by different components, including 1) epigenetic effects, 2) SNP direct effects, 3) environmental effects, and 4) SNP-interaction effects as described in the *Methods* section. Note that in (4), we added the SNP-interaction effect into phenotype simulation to mimic the nonlinear effects. To evaluate the power gained from inclusion of rare variants, the simulation tool can select the proportion of variants to be rare. Phenotypes were simulated based on different linkage disequilibrium (LD) blocks, incorporating both common and rare variants. Various genetic architectures were considered, with different proportions of rare and common variants and different proportions of epigenetic and SNP direct effects to simulate scenarios where regulatory elements influence gene expression and subsequently disease risk. Following other PRS methods (20), simulation studies used real genetic data from the UK Biobank European ancestry samples (N = 20,000) in 2 LD blocks: chr6:192074807-21684054 and chr6:31571218-32682664. Phenotypes were generated as a linear combination of the above four types of effects with added noise as explained in *Methods* section.

The simulations were used to evaluate Epi-PRS as well as several traditional PRS methods, including: 1) LDpred2: A widely used Bayesian approach for PRS estimation. 2) PRS-CS: Another linear-model-based method that utilizes continuous shrinkage priors to account for LD and effect size heterogeneity. 3) Genotype-GBRT: To evaluate whether nonlinear models can improve prediction, we also implemented a Gradient Boosting Regression Trees (GBRT) method to predict disease risk from all variants in the genotype. 4) Genotype-PCA-GBRT: Performing Principal Component Analysis (PCA) first for all the variants in genotype, then using the PCA output as input for GBRT. The PRS-CS and LDpred2 are linear models that rely on GWAS summary statistics, while Genotype-GBRT and Genotype-PCA-GBRT are nonlinear models based on all genetic markers with/without PCA dimension reduction. Since the causal SNPs are located in specific LD blocks, LD block-specific prediction was performed and two LD blocks were selected for testing and comparison of the methods: chr6:192074807-21684054 and chr6:31571218-32682664. The predictive accuracy of the models was assessed using the Area Under the Receiver Operating Characteristic Curve (AUC).

#### The comparison between linear models and nonlinear models.

To quantify the potential increase in power from the inclusion of nonlinear models, a series of simulation experiments varying the number of individuals in the training dataset were conducted. The training dataset sizes were incremented from 1,000 to 2,000, 4,000, 8,000, and 16,000, while the performance was consistently evaluated using a fixed test dataset of 4,000 individuals. In this study, Epi-PRS, Genotype-GBRT, and Genotype-PCA-GBRT were categorized as nonlinear models, whereas PRS-CS and LDPred2 were categorized as linear models. In the simulations, the phenotypes will be affected by the SNPs’ direct effect and SNP-interaction effects while the proportion of epigenetic effects and environmental effects is set to zero. The proportion of rare variants is set to zero, which are the standard simulation setting in previous PRS methods.

**Fig. 2.**
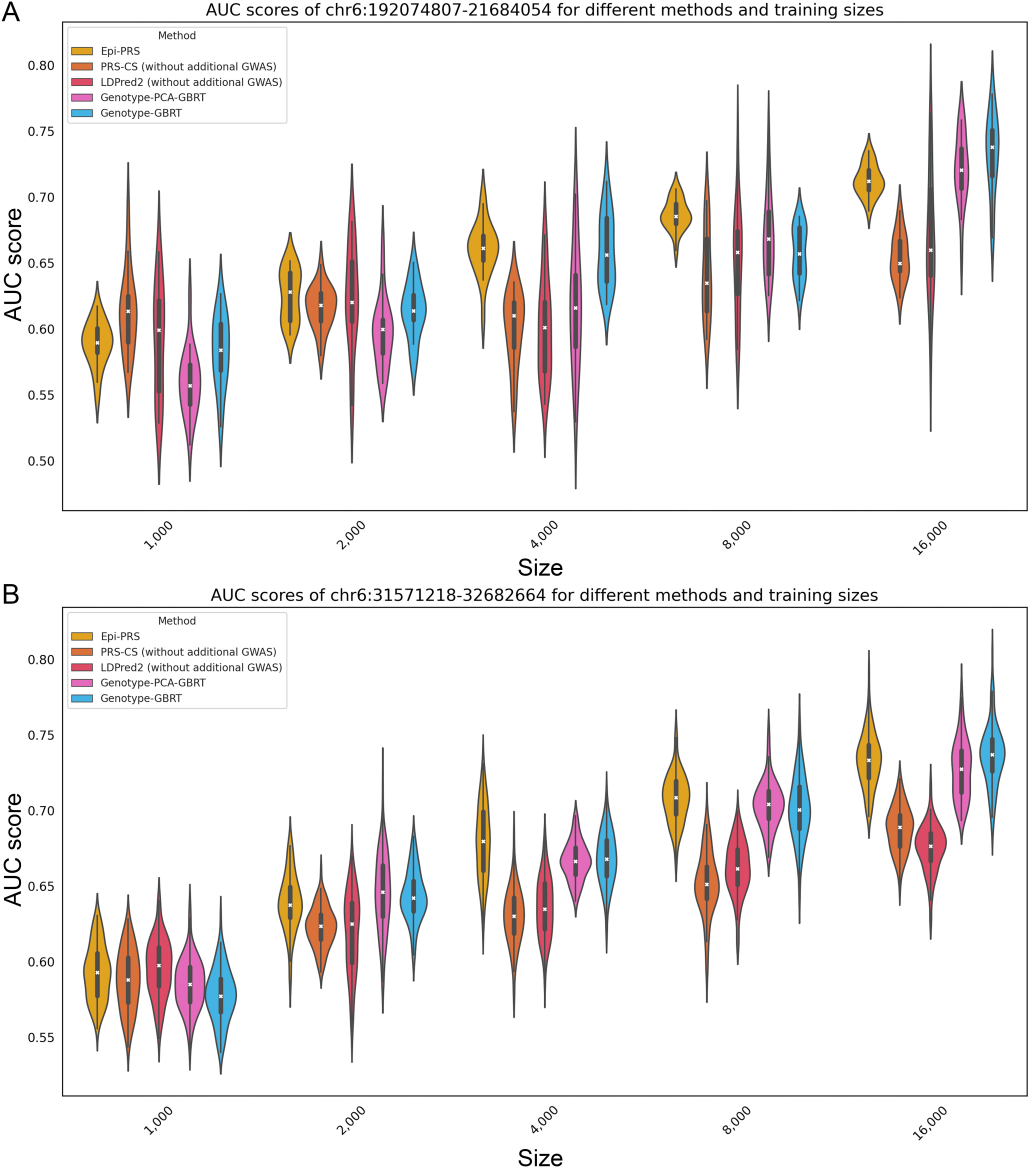
AUC scores for different methods in two LD regions (*A*) chr6:192074807-21684054 and (*B*) chr6:31571218-32682664 with varying numbers of training data. The violin plot in different color represents the AUC score distribution of a specific method based on the 20 independent simulations. It is seen that the performance of different methods increases when the size of training data also increases. No-linear methods (Epi-PRS, Genotype-PCA-GBRT, and Genotype-GBRT) offer significant advantage over linear methods (LDPred2 and PRS-CS) in large sample size settings.

Compared with the traditional linear model, we observed a notable gain in power using the nonlinear framework, with comparable improvements across larger sample sizes (Fig. 2 and *SI Appendix*, Tables S1 and S2). However, when the training dataset is small, nonlinear models tend to overfit, resulting in lower performance compared to linear models. This is likely due to the complexity and flexibility of nonlinear models, which can capture intricate SNP-interaction effects but may overfit when there are insufficient training instances. As the number of training instances increased, the performance of both linear and nonlinear models improved. However, the improvement for nonlinear models was more significant compared to linear models. In particular, when the training dataset exceeded 2,000 instances, nonlinear models began to outperform linear models significantly as we added the nonlinear effect in phenotype simulation. These results indicate that for larger cohorts, nonlinear models are more effective and can achieve superior predictive performance. Conversely, in scenarios with limited training data, linear models exhibit better generalization and are less prone to overfitting. Therefore, the choice between linear and nonlinear models should be guided by the size of the available training dataset. For extensive datasets, nonlinear models are preferable, whereas for smaller datasets, linear models offer better generalizability and reliability.

#### The comparison between whether regulatory information is considered or not.

**Fig. 3.**
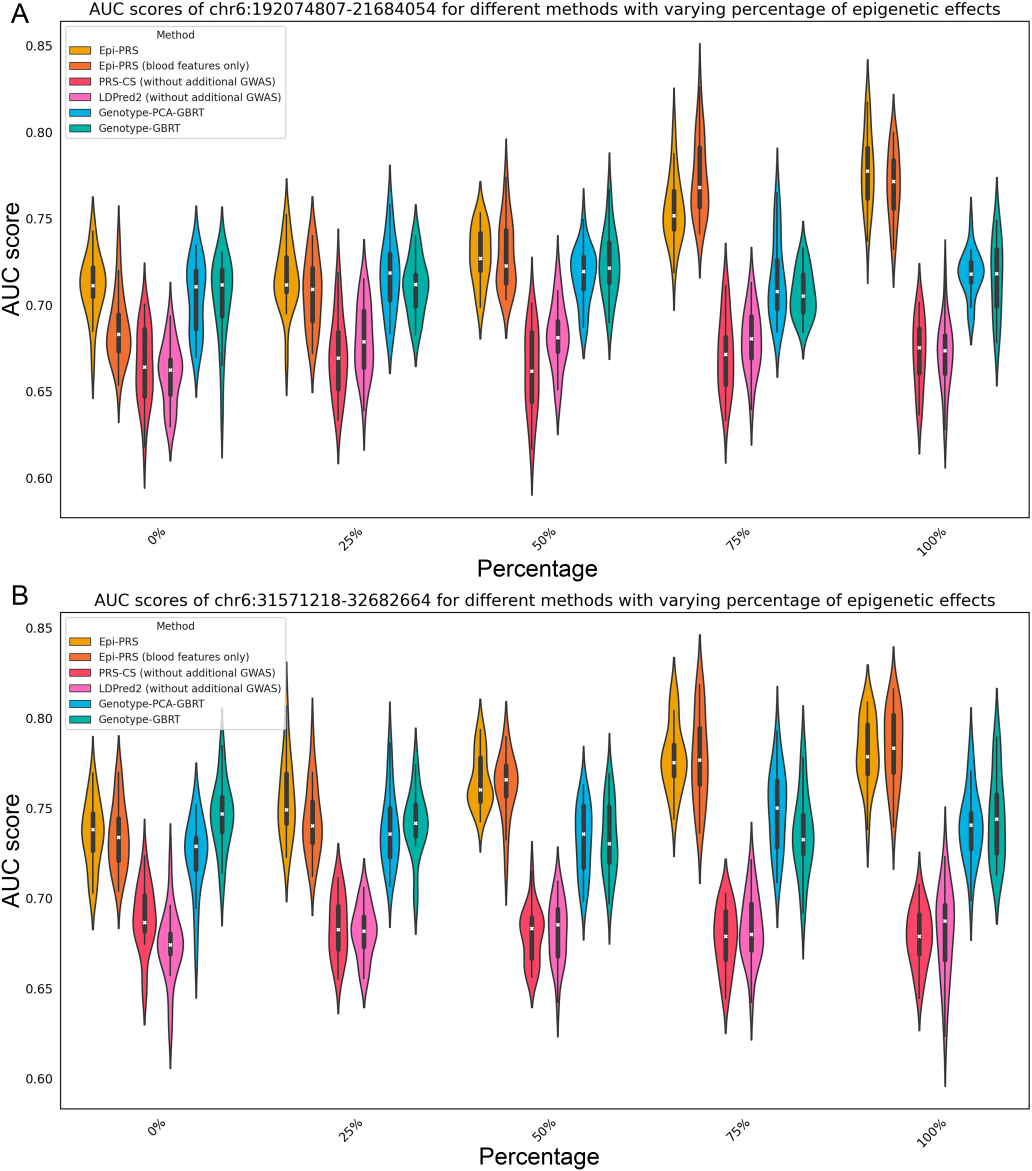
AUC scores for different methods in two LD regions (*A*) chr6:192074807-21684054 and (*B*) chr6:31571218-32682664 with varying percentages of epigenetic effects. The violin plot in different color represents the AUC score distribution of a specific method based on the 20 independent simulations. Epi-PRS offers significant improvement over other methods, especially when the epigenetic effects are large. Reducing dimension of the imputed epigenomic features by only selecting the tissue-specific features leads to notable performance improvement when the disease-related cell types or tissues are known.

To quantify the potential power gain from considering the effect of regulatory mechanisms, a series of simulation experiments with various proportions of epigenetic effects and fixed proportion of SNP interaction effects were conducted. The proportions of epigenetic effects were varied from 0%, 25%, 50%, 75%, and 100% while the proportion of environmental effects and rare variants was set to 0%. Epi-PRS effectively captured the influence of regulatory mechanisms on gene expression and demonstrated better predictive accuracy by achieving a significantly higher AUC compared to LDpred2 and PRS-CS across all simulation scenarios for the two LD blocks (Fig. 3 and *SI Appendix*, Tables S3 and S4). When the epigenetic effect was set to 0% and the SNP direct effect was set to 100%, Epi-PRS, Genotype-GBRT, and Genotype-PCA-GBRT achieved similar AUCs and clearly outperformed traditional PRS methods. On the other hand, there was a clear improvement in the performance of Epi-PRS as the proportion of the epigenetic effect increased, while such improvement was not seen in all the other methods. This suggests that the biggest power gain within the case of the SNPs affecting phenotypes is from epigenetic effect and Epi-PRS can effectively extracting useful regulatory features. When the epigenetic effects were simulated based on regulatory elements in blood cell types, Epi-PRS using blood features (i.e., using only the Enformer predictions for epigenomic data tracks in blood cell types in the training data) further improved the performance compared to Epi-PRS using all features, implying that reducing feature dimension with tissue-specific features is crucial when the disease-related cell types or tissues are known. In contrast, using PCA to reduce the dimension of genotype did not significantly impact the final prediction. These simulation results show that when a disease is affected by epigenetic regulation, Epi-PRS achieves much better performance than existing PRS methods. Furthermore, Epi-PRS power gains are expected to be more dramatic when a better understanding of the disease mechanisms is known and related epigenomic features can be selected.

#### Comparison results under increasing level of contribution by rare variants.

**Fig. 4.**
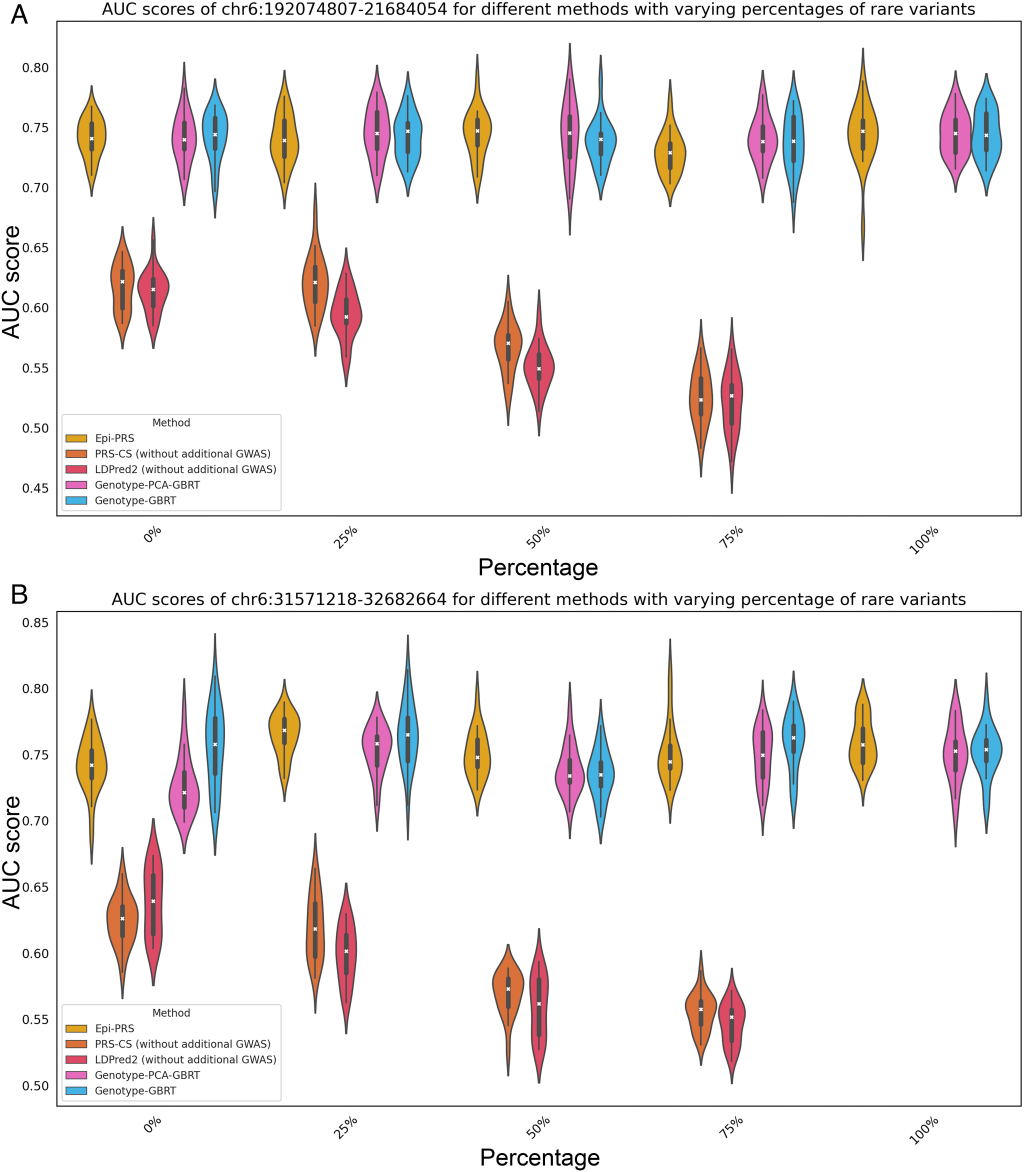
AUC scores for different methods in two LD regions (*A*) chr6:192074807-21684054 and (*B*) chr6:31571218-32682664 with varying percentages of rare variants. The violin plot in different color represents the AUC score distribution of a specific method based on the 20 independent simulations. Performance of linear methods (LDPred2 and PRS-CS) significantly decreases as the percentage of rare variants increases.

Next, the PRS prediction performance was investigated by varying the proportion of rare variants while setting the proportion of epigenetic effects to zero. Epi-PRS again demonstrated better performance by achieving a 12% higher AUC compared to traditional PRS methods across all simulation scenarios (Fig. 4 and *SI Appendix*, Tables S5 and S6). In this setting, the inter-SNP interaction effect was added to simulate the phenotype, a nonlinear effect that cannot be captured by linear models. When the proportion of rare variants was set to 0%, Epi-PRS outperformed traditional methods by capturing nonlinear effects, leading to an increase in AUC by approximately 11 to 12%. However, since the simulation was not related to regulatory information, Epi-PRS performed very similarly to Genotype-GBRT and Genotype-PCA-GBRT. As the proportion of rare variants increased, the performance of Epi-PRS improved, while traditional PRS methods (PRS-CS and LDpred2) decreased, and the Genotype-GBRT and Genotype-PCA-GBRT maintained a similar performance level. In scenarios with 75% rare variants, Epi-PRS showed a remarkable improvement in predictive accuracy, with AUC values increased by up to 20% compared to traditional methods, highlighting its ability to leverage rare variant information effectively. As the proportion of rare variants increased to 100%, the traditional methods failed to predict disease risk, while Epi-PRS consistently performed well.

In the above evaluations, performance was assessed by varying one condition at a time. To further compare the three nonlinear methods, Epi-PRS, Genotype-GBRT, and Genotype-PCA-GBRT were evaluated under combined conditions (50% rare variants and 50% epigenetic effect). The results showed that Epi-PRS achieved an average area under the receiver operating characteristic curve (auROC) of 0.7492, outperforming Genotype-GBRT and Genotype-PCA-GBRT, which had auROCs of 0.6993 and 0.7015, respectively. These comprehensive simulation studies highlight the potential of advanced PRS methods like GBRT and Epi-PRS to significantly advance polygenic risk prediction. In particular, Epi-PRS is anticipated to excel in situations where a significant portion of heritability is primarily driven by rare variants through regulatory effects.

### PRS Prediction on the UK Biobank.

The Epi-PRS method was applied to the prediction of breast cancer and type 2 diabetes (T2D) using data from the UK Biobank. The performance of a predictor was measured by its relative proportional gain in auROC over random guessing, defined as λ=(A-0.5)/0.5, where A is the auROC of the predictor in the test data. This performance index varies between 0 (for a random guess predictor) and 1 (for a perfect predictor).

#### Breast cancer.

Data were collected from 10,547 breast cancer female subjects (cases) and 10,547 female subjects (controls). Five LD blocks were selected based on a significance threshold applied to variants with a stringent *P*-value. Since WGS data are not yet available for all subjects, phased SNP genotype data were used to construct the input genomic sequences for epigenomic feature prediction.

**Fig. 5.**
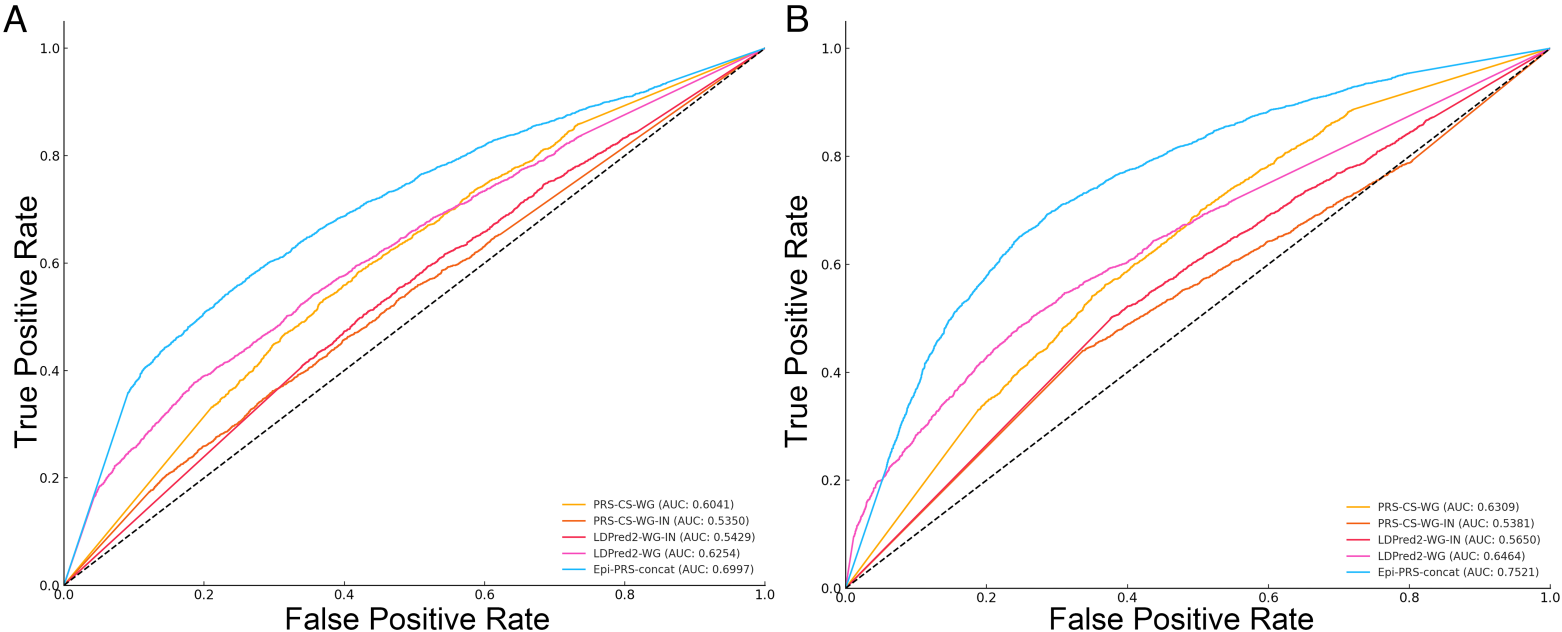
ROC curves of different methods using (*A*) breast cancer and (*B*) type 2 diabetes data from the UKBB. Epi-PRS-concat (combining information from multiple LD blocks) outperforms existing methods that utilize whole genome-wide information and external GWAS summary statistics data.

Initially, the phenotype prediction performance was tested using a single LD block. The dataset was randomly split into an 80% training set and a 20% testing set. The performance of Epi-PRS was compared to three state-of-the-art baseline methods: 1) Genotype-GBRT (using both common and rare variants), 2) LDpred2 (a Bayesian logistic regression method based on common SNP genotypes using external data, such as summary statistics from external GWAS studies using approximately 200,000 samples), and 3) PRS-CS (another Bayesian logistic regression method based on SNP genotypes that also utilized external data). As shown in Table 1, Epi-PRS achieved the best performance in each of the five LD blocks, with an auROC ranging from 0.5379 to 0.5917. The improvement was particularly substantial in the fourth LD block. Next, the information from the five LD blocks was combined by concatenating their epigenomic features. The resulting method, “Epi-PRS-concat,” was compared to four baseline methods: 1) LDpred2-WG (applied to the whole genome), 2) PRS-CS-WG (applied to the whole genome), 3) PRS-CS-WG-IN (based on SNPs from the whole genome without using external GWAS summary statistics), and 4) a combined model. Epi-PRS based on SNPs in the five LD blocks achieved a λ of 0.3994, while the other baseline methods using SNPs from the whole genome achieved λ values between 0.093 and 0.2798 (Fig. 5*A*). The gap between PRS-CS-WG and PRS-CS-WG-IN suggests that the relatively strong performance of PRS-CS-WG is probably due to the extra information provided by external GWAS data.

**Table 1.** The performance of Epi-PRS and baseline methods on polygenic prediction of breast cancer based on single LD block

LD block	PRS-CS	LDPred2	Genotype-GBRT	Epi-PRS
chr5:55417349-56621102	0.5306	0.5194	0.5239	0.5412
chr10:123231465-123900545	0.5445	0.5479	0.5496	0.5554
chr11:68005825-69516130	0.5157	0.5129	0.5168	0.5392
chr16:52035823-5338257	0.5387	0.5341	0.5316	0.5917
chr22:27834752-29651799	0.5049	0.5082	0.5204	0.5497

The performance of Epi-PRS and baseline methods on polygenic prediction of breast cancer based on single LD block. The AUC of different methods using five different LD blocks on breast cancer. Epi-PRS based on only SNPs in the 5 LD blocks achieves a λ of 0.3994 while the other 4 baseline methods using SNPs in the whole genome only achieve λ of 0.093 to 0.2798. λ = (A-0.5)/0.5, where A is the auROC of the predictor in the test data.

#### T2D.

Data were collected from 20,000 case subjects and an equal number of randomly selected control subjects without T2D. Eleven LD blocks were chosen based on a stringent *p*-value cutoff. Epi-PRS with a single LD block (chr16:53382572-55903774) achieved nearly the best performance compared to four baseline methods that utilized information from all variants across the genome (Table 2). Combining information from multiple LD blocks further improved the prediction performance of Epi-PRS, achieving a λ of 0.515, outperforming the other four baseline methods with λ values ranging from 0.077 to 0.3236 (Fig. 5*B*). This nonlinear approach to risk prediction based on epigenomic features significantly improved performance over the baseline methods.

**Table 2.** The performance of Epi-PRS and baseline methods on polygenic prediction of T2D based on single LD block

LD block	PRS-CS	LDPred2	Genotype-GBRT	Epi-PRS
chr3:185068255-186890344	0.5416	0.5235	0.5333	0.5467
chr4:5502388-6773043	0.5387	0.5239	0.5315	0.5548
chr6:19207487-21684065	0.5247	0.5175	0.5386	0.5721
chr6:31571218-32682664	0.5187	0.5129	0.5474	0.6083
chr6:32682664-33236497	0.5257	0.5166	0.5447	0.5574
chr8:116096495-119685457	0.5253	0.5012	0.5287	0.6072
chr9:20463534-22206559	0.5327	0.5281	0.5354	0.5894
chr10:93335047-95396368	0.505	0.5052	0.5218	0.5530
chr10:112561493-115328432	0.5604	0.5189	0.5537	0.6229
chr12:3677037-4417679	0.5235	0.5191	0.5343	0.5432
chr16:53382572-55903774	0.5045	0.5067	0.5322	0.6543

The AUC of different methods using eleven different LD blocks. Epi-PRS achieves a λ of 0.515 while other 4 baseline methods only achieve λ of 0.077 to 0.3236. λ = (A-0.5)/0.5, where A is the auROC of the predictor in the test data.

Our results demonstrate that incorporating haploid sequence context around each variant and accounting for personal variant effects in diverse cellular environments is a powerful strategy for improving PRS prediction. Epi-PRS outperformed traditional PRS methods in both breast cancer and T2D, where regulatory mechanisms play a crucial role. By integrating epigenomic features from multiple cellular contexts, Epi-PRS provides a more comprehensive assessment of genetic risk, highlighting its potential to enhance polygenic prediction and deepen our understanding of complex disease genetics.

To assess the robustness of Epi-PRS in the presence of confounding factors, we conducted additional analyses on geographical stratification, which can introduce complexities related to population structure and regional variations in genetic and environmental factors. Using breast cancer as a case study, we partitioned the training and testing sets by region—training on individuals from England and Wales and testing on individuals from Scotland. The model achieved an AUC of 0.6874 under geographically stratified sampling, compared to 0.6997 under random partitioning, indicating that Epi-PRS remains robust even in the presence of population structure differences. This underscores the model’s generalizability and its ability to account for real-world confounding effects. To further strengthen Epi-PRS, we plan to explicitly incorporate confounders into the modeling process, ensuring that predictions remain biologically meaningful, interpretable, and unbiased.

Additionally, Epi-PRS achieves its superior performance with a limited number of LD blocks (5 for breast cancer, 11 for T2D), suggesting that further expansion could further improve predictive accuracy. To test whether Epi-PRS falsely detects signals, we randomly sampled three LD blocks without significant SNPs. These blocks had a mean AUC of 0.5026—close to random—demonstrating that Epi-PRS does not spuriously detect signals (*SI Appendix*, Table S7). And combining the weakest 3 LD blocks achieved an AUC of 0.5078. In contrast, Epi-PRS with the 5 strongest signal LD blocks achieved an AUC of 0.6997, reinforcing its ability to distinguish true associations from noise. We also conducted a leave-one-LD-out analysis across the 11 LD blocks used in our polygenic prediction of T2D. Despite some individual LD blocks achieving high predictive performance when used in isolation, the overall AUC for Epi-PRS remained consistently high across the genome when evaluated using a leave-one-block-out strategy. Specifically, AUC values ranged from 0.7197 to 0.7421, reflecting stable predictive accuracy with only modest variation across different exclusion scenarios.

To further evaluate the biological interpretability of Epi-PRS, we performed additional analyses focusing on feature importance and bin importance. We investigated the importance of epigenomic signals and genomic bins using the LD block on chromosome 16 associated with T2D as a representative example. At the feature level, among the top 10 most informative epigenomic features, 4 were derived from pancreas or liver tissues. When expanded to the top 50 features, 22 (44%) originated from these tissues, indicating a strong enrichment of regulatory information from disease-relevant cellular contexts. At the bin level, we compared the top and bottom 1% of bins (197 bins each) based on feature importance scores. Among the top 1% bins, 87.82% (173/197) overlapped with open chromatin regions identified by DNase-seq in at least one of ten liver or pancreas datasets (across fetal and adult samples). In contrast, only 9.13% (18/197) of the bottom 1% bins exhibited DNase signal. Furthermore, 81% (140/173) of the DNase-positive high-importance bins also overlapped with H3K27ac-marked active regulatory regions in the same tissues. These results suggest that Epi-PRS places significant weight on bins corresponding to active enhancers and promoters in biologically relevant tissues.

Taken together, these results establish Epi-PRS as a robust and interpretable approach to polygenic prediction, capable of leveraging regulatory insights to enhance disease risk assessment. We hope to expand Epi-PRS to effectively handling real-world confounders in the future.

## Discussion

With the increasing availability of WGS data, significant challenges and opportunities arise in the field of genetics for improving disease risk prediction. To address these challenges, this study introduces Epi-PRS, an approach that explores several potential improvements to existing PRS methods. These improvements include 1) the adoption of nonlinear models: nonlinear models have the potential to capture complex interactions between genetic variants that linear models may not detect. By leveraging these nonlinear models, the accuracy and predictive power of PRS can potentially be enhanced. Simulation studies in this research demonstrated that nonlinear models, such as Epi-PRS, outperform linear models when the training dataset is sufficiently large. This finding suggests that nonlinear models are more effective in capturing intricate genetic interactions and can provide superior predictive performance for large cohorts; 2) the consideration of rare variants: rare genetic variants, despite their low frequency, can have substantial impacts on disease risk. Incorporating these variants into PRS calculations can provide a more comprehensive and accurate assessment of genetic risk. Epi-PRS achieves this by doing risk modeling based epigenomic features predicted by a genomic LLM that can integrate information from all the variants, including rare or de novo variants, on the two sequence alleles of a genetic loci. The simulation studies indicated that Epi-PRS may significantly benefit from including rare variants, particularly in scenarios where rare variants play a critical role in disease etiology. In contrast, genotype-based PRS methods, even those using advanced nonlinear predictions, may miss the contributions of rare variants, leading to suboptimal predictions, and 3) the integration of regulatory information: regulatory elements play a critical role in gene expression and disease manifestation. By integrating regulatory information into PRS models, deeper insights into the genetic architecture of diseases can be gained, potentially improving the precision of risk predictions. The simulation studies confirmed the importance of considering regulatory mechanisms. This result underscores the value of integrating epigenomic features to enhance the predictive accuracy of PRS models.

The comprehensive simulation studies conducted in this research systematically evaluated the impact of nonlinear models, rare variants, and regulatory information on PRS prediction. These evaluations provide valuable insights into how each aspect contributes to the performance of PRS methods. By addressing the limitations of existing PRS methodologies, the findings suggest that significant advancements in disease risk modeling can be achieved, ultimately enhancing the understanding of precision medicine. To test Epi-PRS in real data, we apply it to breast cancer and type 2 diabetes data from the UK Biobank. The results, based on personal genome sequences imputed from genotyping array data, showed that Epi-PRS outperformed existing PRS methods, confirming the potential for improved disease risk prediction through the integration of nonlinear models, rare variants, and regulatory information. This study indicates that incorporating these elements into PRS models can lead to more accurate and comprehensive assessments of genetic risk. Although we were forced to imputed phased genomic sequences because WGS data were not available at the time of this research, the improvement by Epi-PRS over the competing methods should be real. In fact, the improvement would have been even larger if Epi-PRS were based on the real WGS data for the subjects.

While the results of this study are promising, further validation across different diseases is essential to ensure the broad applicability and effectiveness of Epi-PRS. We compared the Spearman’s correlation between the predicted PRS for different methods in *SI Appendix*, Fig. S1. These results reveal that the Spearman’s correlation coefficients between methods are relatively low, suggesting notable differences in how each method ranks individuals based on genetic risk. Notably, Epi-PRS relies on Enformer annotations without directly incorporating SNPs into the prediction model. While this approach captures regulatory effects mediated through transcriptional regulation, it may not account for genetic effects that act through protein-coding sequences, RNA processing, translation, or splicing. This potential information loss raises the question of whether the best predictive performance arises from genotype-only models, epigenomic feature-only models, or a combination of both, depending on the disease of interest. Future research should focus on evaluating the relative contributions of predicted epigenomic features and direct genetic features and on developing predictive models capable of effective usage of different types of features. Additionally, the integration of other LLMs in place of Enformer could offer further improvements. Future models might provide faster inference, enabling more efficient data processing (38, 39). For example, QUICK can be adopted as they use a group of novel optimized CUDA kernels for the efficient inference of quantized LLMs (40). Another improvement is the high-resolution predictions of epigenomic and genomic profile. By accurately predicting the impact of genetic variants on transcription factor binding at a single base-pair resolution, BPNet enhances the precision of transcription factor binding prediction (41). This model can identify how individual variants alter the binding affinity of transcription factors, offering detailed insights into gene regulation mechanisms. Furthermore, developing models that incorporate cellular context specificity will allow for more accurate modeling of disease-specific regulatory mechanisms. For instance, models like EpiGePT have been designed to predict various types of epigenomic signals given a specific cellular context (42). By integrating such models, we can better understand how genetic variants influence disease risk in a context-dependent manner. This is crucial for diseases that manifest differently across various cell types and tissues, such as cancers and autoimmune disorders. While the current Epi-PRS utilizes pretrained LLMs and applies dimension reduction to epigenomic features for computational efficiency, it is essential to develop more interpretable LLM-based methods capable of identifying causal SNPs and epigenomic signals. This advancement will be a critical step toward enhancing the real-world utility of PRS.

In summary, this research underscores the importance of integrating advanced computational models, rare variant analysis, and regulatory information to improve PRS methods. These innovations have the potential to elevate the precision of genetic risk predictions and facilitate a deeper understanding of the regulatory mechanisms underlying complex diseases. As a result, the goal of personalized medicine, where treatments and preventive strategies are tailored to the unique genetic makeup of each individual, becomes more attainable.

## Materials and Methods

### Workflow of the Epi-PRS.

This workflow is composed of three major steps: 1) personal genome construction, 2) epigenomic feature extraction, and 3) risk prediction, which are detailed as follows.

#### Personal genome construction.

The first step in the Epi-PRS workflow involves constructing the maternal and paternal genomes from personal WGS data, which serve as the input for Epi-PRS. This process is detailed in the following steps: A) Variant Filtering: The current version of Epi-PRS focuses exclusively on SNVs. We start by processing the variant call format (VCF) file containing the genetic profiles of all individuals. Using vcftools (43), we filter out all insertions and deletions, retaining only the SNPs. B) Genotype Phasing: Next, we phase the genotypes using the reference-free Beagle (44) software. Phasing is essential for distinguishing between the maternal and paternal alleles, which is critical for subsequent steps. C) Personal Genome Construction: Finally, we employ the vcf2diploid (45) tool to reconstruct the maternal and paternal personal genomes for each individual. This step produces two separate haploid genome sequences for each person, representing the maternal and paternal contributions.

#### Epigenomic feature extraction.

In the second step, we extract epigenomic features using a pretrained genomic LLM, such as Enformer. This process involves several substeps: A) Feature Extraction: The genomic LLM (e.g., Enformer) is applied to each haploid genome to predict genomic and epigenomic features, including gene expression, chromatin accessibility, ChIP-seq signals, and histone modification signals across a diverse set of cell lines and tissues. Taking Enformer as the feature extractor, for each input DNA sequence of 196,608 bp, the Enformer model generates an d=5,313-dimensional feature vector for each of the central nonoverlapping k=896 bins, each with a bin size of 128 bp. B) Sliding Window Approach: Given that a single linkage disequilibrium (LD) block is typically larger than the input length for Enformer, we utilize a sliding window approach within each LD block to capture features from multiple input regions. If an LD block contains l input regions, the extracted features for this block will have dimensions of (l,k,d). We denote these features as $\left\{x_{i}^{m},x_{i}^{p},i=1,\cdots 896\right\}$, where the superscript indicates the parent of origin (m for maternal and P for paternal). C) Dimension Reduction: To manage the high dimensionality of the extracted features, we apply local PCA to each bin across individuals. This dimension reduction step reduces the features to a more manageable size, denoted as $d^{\prime }$ where $d^{\prime }\ll d$.

#### Risk prediction.

The final step involves building predictive models for disease risk based on the reduced-dimension features and the phenotypic data: A) Model Construction: For binary classification tasks, such as disease presence or absence, we construct a GBRT classification model. For continuous traits, we use GBRT regression model. These models are trained on the individual features obtained from the feature extraction step. B) Training and Testing: We randomly select 80% of both case and control subjects as the training set, leaving the remaining 20% as the testing set. The performance of the models is evaluated using prediction results on the testing set. C) Performance Evaluation: The predictive accuracy of the models is assessed using metrics such as the AUC for classification tasks and the coefficient of determination (R^2^) for regression tasks. These metrics provide insight into the effectiveness of Epi-PRS in predicting disease risk based on integrated genomic and epigenomic data.

### Genomic LLM.

Different from previous convolutional and recurrent neural network models, recent developments in LLMs typically rely on the transformer architecture, which was introduced by Vaswani et al. (46) as a revolutionized deep learning method, offering more efficient training and better handling of long-range dependencies in sequential data. The vanilla transformer model is divided into two main components: the encoder and the decoder, both of which share a similar basic architecture composed of a stack of identical blocks (47). Each block in the transformer model consists of two key sublayers: 1) Multi-Head Attention Sublayer: This sublayer allows the model to attend to different positions of the input sequence simultaneously, capturing various aspects of the data. It computes attention scores in multiple parallel heads, providing diverse perspectives on the input data. These scores are then aggregated to form a comprehensive understanding of the sequence. 2) Feed-Forward Sublayer: This sublayer consists of fully connected feed-forward networks applied independently to each position in the sequence. It includes a nonlinear activation function, typically ReLU, which helps in capturing complex patterns and interactions within the data. Both sublayers are followed by layer normalization, which standardizes the inputs to each layer, improving the stability and performance of the model. Additionally, a residual connection (48) around every sublayer is applied in each block to help mitigate the vanishing gradient problem. These residual connections add the input of the sublayer to its output, ensuring that the gradients can flow through the network more effectively during backpropagation.

Our previous review (49) describes each module and layer that constitutes the transformer model in detail, exploring their mechanisms and potential applications in bioinformatics. In this paper, we adopt Enformer (50), one of the most advanced genomic LLMs, as our default choice of LLM. Enformer innovatively integrates the transformer encoder structure to predict 5,313 epigenomic signals in humans, significantly enhancing its capabilities over previous CNN-based models. Enformer brings several improvements that make it particularly well-suited for genomic data: 1) Extended Receptive Field: The transformer encoder structure in Enformer greatly increases the receptive field of the network to 196,608 bp. This extensive receptive field allows the model to capture long-range interactions and dependencies within the genomic sequence, which are crucial for understanding complex regulatory mechanisms and gene expression patterns. 2) Improved Predictive Performance: By leveraging the transformer architecture, Enformer achieves superior predictive performance in estimating a wide array of epigenomic signals, including chromatin accessibility, transcription factor binding, and histone modifications. These improvements make Enformer highly effective in modeling the intricate relationships between genetic variants and molecular phenotypes.

Overall, the adoption of Enformer in our study exemplifies the significant advancements in genomic LLMs, enabling more accurate and comprehensive predictions of epigenomic features from genomic sequences. This innovation is a key component of the Epi-PRS framework, which leverages these detailed molecular phenotype predictions to enhance disease risk prediction and improve our understanding of the genetic architecture of complex traits.

### Dimension Reduction of Genomic LLM Features.

For each 196,608 bp input region, the Enformer-based method generates a substantial number of predicted features—approximately 9.5 million (896 bins × 5,313 features per bin × 2 haploid genomes). This high dimensionality presents a significant challenge for risk modeling, as traditional statistical methods struggle to handle such a vast number of predictor variables effectively. To address this issue, we implement local PCA strategies for dimension reduction, which is demonstrated in the four steps below.

1. **Feature Vector Formation**: For the $i^{\mathrm{th}}$ bin, we construct a set of feature vectors {$x_{i}^{m}\left(j\right),x_{i}^{p}\left(j\right),j=1,\cdots ,N\}$ where j indexes all cases or controls in the study, and $x_{i}^{m}\left(j\right)$ and $x_{i}^{p}\left(j\right)$ represent the maternal and paternal feature vectors, respectively. Each feature vector contains 5,313 dimensions, corresponding to the genomic and epigenomic signals predicted by Enformer.
2. **PCA**: PCA is performed on this set of feature vectors to reduce the dimensionality from 5,313 to a much smaller value. This reduction is crucial for making the subsequent risk modeling computationally feasible and statistically robust. For example, we reduce the dimension to 5 principal components, which capture the majority of the variance in the original high-dimensional feature vectors. The detailed comparison for selecting the number of PCs is shown in *SI Appendix*, Table S8. The number of PCs should reflect an optimal balance between information retention, computational feasibility, and model robustness.
3. **Reduced-Dimension Feature Vectors**: After PCA, we obtain a sequence of reduced-dimension feature vectors $\left\{r_{i}^{m},r_{i}^{p},i=1,\cdots 896\right\}$ for each individual in the study. These vectors characterize the epigenomic states of the input region while retaining the most informative aspects of the original data. Here, $r_{i}^{m}$ and $r_{i}^{p}$ are the reduced-dimension feature vectors for the maternal and paternal sequences, respectively, for the $i^{\mathrm{th}}$ bin.
4. **Combining Features Across Bins**: The reduced-dimension feature vectors from all 896 bins are then concatenated to form a comprehensive feature set for each individual. This results in a significantly reduced yet highly informative feature matrix, which captures the essential epigenomic signals across the entire 196,608 bp input region.

By adopting local PCA for dimension reduction, we can manage the high dimensionality of the Enformer-predicted features, ensuring that our risk models are both computationally efficient and statistically robust. This approach allows us to harness the rich epigenomic information provided by Enformer, ultimately leading to more accurate and comprehensive disease risk predictions.

### Selection of Informative LD Block and Application in the UKBB.

In principle, we could include the reduced-dimension features from each approximately 200 kb region tiling the genome as predictor variables in the risk model. However, given that there are about 15,000 such regions across the genome, each contributing 4,480 features (896 bins × 5 principal components), the number of predictor variables could still be prohibitively large. To address this issue, we propose a further dimension reduction strategy by focusing on regions preselected based on the enrichment of GWAS signals for the phenotype of interest. We regard the presence of SNPs with highly significant P-values for association with the disease as an indication that the corresponding LD block contains genetic variations useful for risk modeling.

We can summarize the main steps of our method as follows:

1. **Joint Variant Calling and Phasing**:
  a)  Perform joint variant calling for the individuals in the study based on their WGS data.
  b)  Phase the detected SNVs to obtain haploid genome sequences for each individual, distinguishing maternal and paternal genomes.
2. **GWAS-Based SNP Selection**:
  a)  Utilize prior GWAS studies or perform GWAS analysis using the current dataset to identify a set of SNPs associated with the disease of interest. These SNPs should have *P*-values smaller than a predefined threshold (denoted as $P_{0}$).
  b)  Select all LD blocks that contain SNPs with *P*-values less than $P_{0}$. This ensures that we focus on genomic regions that are most likely to contribute to the disease risk.
3. **Tiling LD Blocks and Feature Extraction**:
  a)  For each selected LD block, tile the block with overlapping 196,608 bp input regions, focusing on the central 114,668 bp region to ensure comprehensive coverage.
  b)  Apply the Enformer model to the maternal and paternal input sequences for these regions to obtain the epigenomic feature vectors $\left\{x_{i}^{m},x_{i}^{p},i=1,\cdots M\right\}$, where M indicates the total number of 128 bp bins required to cover the LD block.
4. **Dimension Reduction of Epigenomic Features**:
  a)  Perform PCA on the feature vectors $x_{i}^{m},x_{i}^{p}$ for each bin to reduce the dimensionality from 5,313 to a smaller value (e.g., 5 principal components).
  b)  Concatenate the reduced-dimension feature vectors $r_{i}^{m},r_{i}^{p}$ across all bins within each LD block to form a comprehensive feature vector for the entire LD block.
5. **Risk Prediction Modeling**:
  a)  Construct a feature vector $x_{b}$ for each selected LD block.
  b)  Concatenate the PCA-projected feature vectors ${\tilde{x}}_{b}$ from all selected LD blocks to form a global feature vector ${\tilde{x}}_{\mathrm{concat}}$.
  c)  Apply GBRT or other binary prediction methods to learn a risk predictor based on ${\tilde{x}}_{\mathrm{concat}}$. Alternatively, fit LD block-specific risk prediction models based on ${\tilde{x}}_{b}$ for each LD block and then learn an optimally weighted combination of these models as the final risk prediction model.
6. **Application to UK Biobank Data**:
  a)  Obtain GWAS summary statistics data from the UKBB, excluding the testing individuals for each disease, to identify the most significant variants for breast cancer and diabetes.
  b)  Apply a stringent *P*-value cutoff of 5e-17 for breast cancer, identifying 89 significant variants, and a cutoff of 5e-20 for diabetes, identifying 3,015 significant variants.
  c)  Intersect these significant variants with 1,702 LD blocks and identify 5 LD blocks for breast cancer and 11 LD blocks for diabetes that contain significant variants.

By integrating GWAS-enriched regions and performing local PCA for dimension reduction, our approach effectively narrows down the predictor variables to the most relevant genomic features. This enables more efficient and accurate risk modeling, leveraging both genotypic and epigenomic information to provide a comprehensive understanding of disease susceptibility.

### Phenotype Simulation.

To systematically evaluate the performance, we aim to simulate phenotypes using real genotype data from the UK Biobank. Since Epi-PRS integrates regulatory information, which is cell-type specific, we develop a regulatory-based phenotype simulation tool called RegPheno that can sample both rare and common variants. RegPheno is freely available under the Epi-PRS GitHub repository. This allows us to control the fraction of rare variants contributing to the disease phenotype, providing a comprehensive evaluation of Epi-PRS across different genetic architectures.

For n individuals, let $y_{i}$ denote a binary trait (1 indicating a case and 0 indicating a control) following the Bernoulli distribution with mean $\mu_{i},i=1,\cdots ,n$. We consider the following generalized linear model:

$$g\left(\mu_{i}\right)=\alpha_{0}+y_{i}^{\mathrm{epi}}+y_{i}^{\mathrm{direct}}+y_{i}^{\mathrm{env}}+y_{i}^{\mathrm{inter}},$$

where g is the logit function, $y_{i}^{\mathrm{epi}},y_{i}^{\mathrm{direct}}$, $y_{i}^{\mathrm{env}}$ and $y_{i}^{\mathrm{inter}}$ denote the epigenetic effect, SNP direct effect, environmental effect, and SNP interaction effect, respectively. $\alpha_{0}$ is determined such that the expected number of cases equals nλ where λ is the prespecified sample prevalence rate and is set to 0.5.

#### Epigenetic effect.

We assume $y_{i}^{epi}=\sum_{g\in G}\sum_{t\in T}\sum_{k\in S_{g}}X_{ik}B_{tk}e_{gtk}$. Here, G is the set of causal genes and we randomly selected one causal gene per LD block. T is the set of expressed transcription factors (TFs) with median reads > 10 in blood tissue from GTEx. $S_{g}$ is the set of causal variants in neighboring regulatory elements (REs) for gene g (distance to the gene body < 1MB). REs are defined as candidate cis-regulatory elements (cCREs) in blood tissue from ENCODE, and we consider 100 causal variants per causal gene throughout the simulations. $X_{\mathrm{ik}}$ denotes the normalized genotype for variant k in individual i. $B_{\mathrm{tk}}$ is a binary variable representing whether variant k overlaps with binding site of TF t where 1 indicates overlap and 0 indicates no overlap. TF binding sites are derived using the motif scanning tool HOMER to scan along the reference genome and match the motifs of each TF. We further assume the random effect term $e_{\mathrm{gtk}}∼N\left(0,\sigma_{t}^{2}\right)$, where $\sigma_{t}^{2}$ follows an inverse gamma distribution IG(3,1). The epigenetic effect $y_{i}^{\mathrm{epi}}$ is then normalized to have mean 0 and variance $h_{\mathrm{epi}}^{2}$ across n individuals. Cases and controls are simulated by thresholding in these n individuals.

#### SNP direct effect.

We assume $yi^{direct}=\sum_{k\notin \cup g\in G^{s_{g}}}X_{ik}\beta_{k}$, where $\beta_{k}∼N\left(0,1\right)$. The SNP direct effect is summed over SNPs that do not overlap any REs of causal genes. Here, we assume that the epigenetic effect is independent of the SNP direct effect, which is convenient for evaluation. The SNP direct effect is further normalized to have mean 0 and variance $h_{\mathrm{direct}}^{2}$ across n individuals.

#### Environment effect.

We assume $y_{i}^{\mathrm{env}}∼N\left(0,1-h_{\mathrm{epi}}^{2}-h_{\mathrm{direct}}^{2}-h_{\mathrm{inter}}^{2}\right)$.

#### SNP interaction effect.

We assume a fraction of the causal SNPs have multiplicative interaction effect (where one SNP increases or decreases the effect of the other).

By combining these effects, we can simulate phenotypes that reflect the complex interplay among three components: 1) genetic variants, 2) regulatory elements, 3) environmental factors, and 4) SNP interactions. Additionally, we can adjust the proportion of rare variants contributing to the disease by selectively sampling these variants during the simulation process. This flexible simulation approach allows us to comprehensively assess the robustness and accuracy of Epi-PRS in various genetic contexts, providing valuable insights into its performance across different scenarios. Note that the Epi-PRS method implicitly assumes a model in which genetic variants affect the phenotype through their effects on epigenomic features. Instead of simulating phenotype based on such a model, we opt to use the above random effect model in order to avoid biasing the comparison in favor of our method. For each setting considered, the simulation was repeated 20 times.

### Feature Importance and Bin Importance.

In the final stage of Epi-PRS, we extracted top PCs derived from 5,313 predicted epigenomic signals (e.g., chromatin accessibility, histone marks, TF binding) across different cell types for each genomic bin. Then PCA features across all bins within an LD block were concatenated and fed to a GBRT classifier. In order to analyze the feature importance of the original 5,313 epigenomic signals instead of PCA features, we assessed feature importance by training a GBRT directly on the 5,313 predicted epigenomic features for each single 128 bp bin for each LD block. Feature importance was computed using the Gini importance metric, and the resulting scores were then averaged across all bins to obtain the overall importance of each epigenomic track.

To assess which specific 128 bp bins within the LD block are most predictive, we computed the Gini importance of each PC feature from the GBRT model. Since each bin is represented by top PCs in the final step of Epi-PRS, we calculated the bin-level importance by averaging the importance scores of its associated PCs. This yielded a bin-level ranking of predictive contribution.

## Supplementary Material

Appendix 01 (PDF)

## Data Availability

Raw data (genotype and phenotype) from the UKBB participants can be requested from the UKBB Access Management System (https://bbams.ndph.ox.ac.uk) (51). Code is publicly available online at https://github.com/SUwonglab/Epi-PRS (52).
