# Supplementary material for: Improving polygenic prediction from whole-genome sequencing data by leveraging predicted epigenomic features
Source: Proc Natl Acad Sci U S A. 2025 Jun 12;122(24):e2419202122. doi: 10.1073/pnas.2419202122 (PMC12184400; doi:10.1073/pnas.2419202122)
Supplement: Supplementary file 1 — Appendix 01 (PDF) [file pnas.2419202122.sapp.pdf]

PNAS

Supporting Information for

Improving polygenic prediction from whole-genome sequencing data by leveraging predicted epigenomic features

Wanwen Zeng[1,4], Hanmin Guo[1,3,4], Qiao Liu[1,4], and Wing Hung Wong[1,2,4,*]

[1] Department of Statistics, Stanford University, Stanford, CA 94305, USA;
[2] Department of Biomedical Data Science, Stanford University, Stanford, CA 94305, USA;
[3] Department of Psychiatry and Behavioral Sciences, Stanford University, Stanford, CA 94305, USA;
[4] Bio-X Program, Stanford University, Stanford, CA 94305, USA;
* To whom correspondence should be addressed

Email: whwong@stanford.edu

This PDF file includes:

Figure S1
Tables S1 to S8

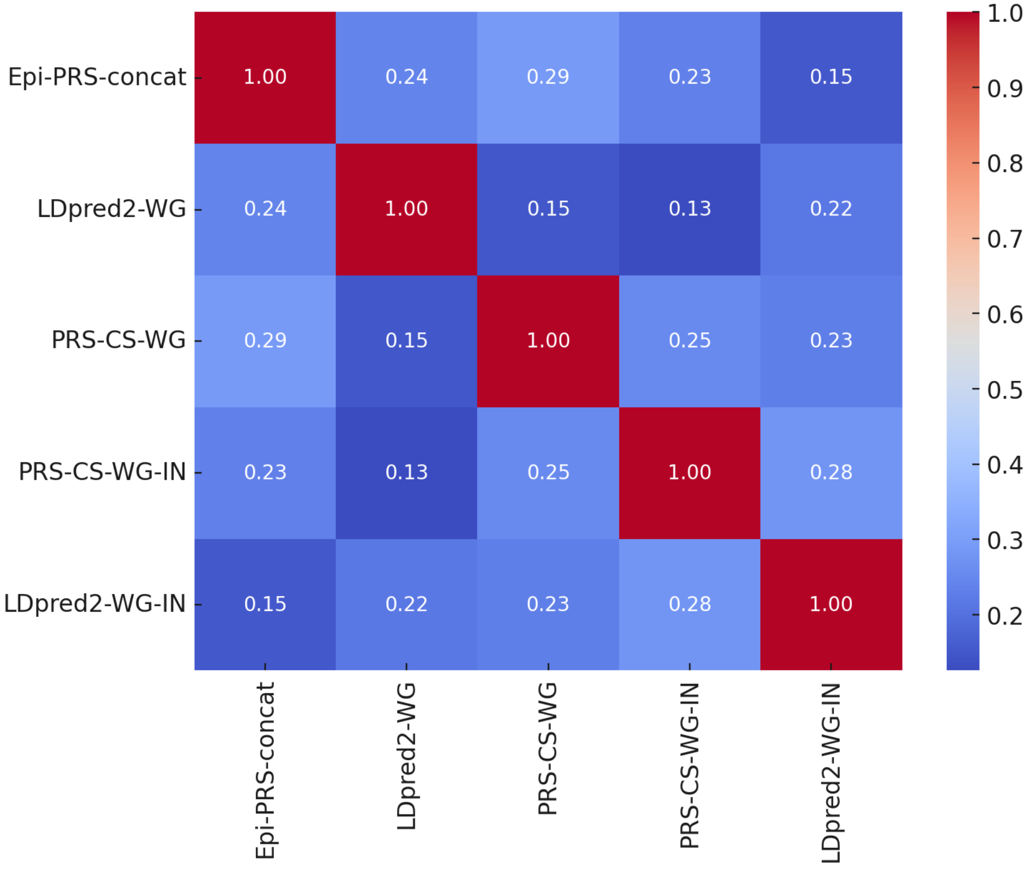

Figure S1. The heatmap for Spearman's correlation between predicted PRS for different methods in breast cancer. These results reveal that the Spearman's correlation coefficients between methods are relatively low, suggesting notable differences in how each method ranks individuals based on genetic risk. This finding indicates that the predictive effects of different PRS approaches may vary substantially at the individual level, likely due to differences in the underlying modeling strategies, inclusion of regulatory features, and methodological assumptions.

Table S1. Simulated Results with Varying the Number of Training Data. This table presents the simulated results for the genomic region chr6:192074807-21684054, highlighting the impact of linear and non-linear models with varying training data set. The evaluation metric used is the average AUC score, calculated over 20 repetitions. In these simulations, the proportion of epigenetic effects and the propitiation of rare variants are set to zero.

**chr6:192074807-21684054**	**1,000**	**2,000**	**4,000**	**8,000**	**16,000**
Epi-PRS	0.5886 ± 0.0167	**0.6256 ± 0.0190**	**0.6605 ± 0.0196**	**0.6866 ± 0.0112**	0.7126 ± 0.0115
PRS-CS (without additional GWAS)	**0.6127 ± 0.0301**	0.6160 ± 0.0157	0.6000 ± 0.0273	0.6421 ± 0.0329	0.6548 ± 0.0175
LDPred2 (without additional GWAS)	0.5912 ± 0.0411	0.6230 ± 0.0387	0.5999 ± 0.0356	0.6511 ± 0.0397	0.6692 ± 0.0465
Genotype-PCA-GBRT	0.5593 ± 0.0242	0.5971 ± 0.0255	0.6171 ± 0.0448	0.6707 ± 0.0308	0.7213 ± 0.0259
Genotype-GBRT	0.5828 ± 0.0267	0.6139 ± 0.0202	0.6595 ± 0.0268	0.6578 ± 0.0193	**0.7324 ± 0.0291**

Table S2. Simulated Results with Varying the Number of Training Data. This table presents the simulated results for the genomic region chr6:31571218-32682664, highlighting the impact of linear and non-linear models with varying training data set. The evaluation metric used is the average AUC score, calculated over 20 repetitions. In these simulations, the proportion of epigenetic effects and the propitiation of rare variants are set to zero.

**chr6:31571218-32682664**	**1,000**	**2,000**	**4,000**	**8,000**	**16,000**
Epi-PRS	0.5915 ± 0.0169	0.6390 ± 0.0184	**0.6775 ± 0.0213**	**0.7062 ± 0.0191**	0.7360 ± 0.0172
PRS-CS (without additional GWAS)	0.5919 ± 0.0220	0.6220 ± 0.0121	0.6316 ± 0.0189	0.6528 ± 0.0209	0.6899 ± 0.0142
LDPred2 (without additional GWAS)	**0.5975 ± 0.0204**	0.6208 ± 0.0265	0.6349 ± 0.0227	0.6625 ± 0.0172	0.6761 ± 0.0153
Genotype-PCA-GBRT	0.5860 ± 0.0168	0.6426 ± 0.0252	0.6664 ± 0.0126	0.7047 ± 0.0171	0.7249 ± 0.0193
Genotype-GBRT	0.5815 ± 0.0185	**0.6448 ± 0.0184**	0.6679 ± 0.0175	0.7015 ± 0.0208	**0.7392 ± 0.0209**

Table S3. Simulated Results with Varying Proportions of Epigenetic Effects. This table presents the simulated results for the genomic region chr6:192074807-21684054, highlighting the impact of different proportions of epigenetic effects on prediction performance. The evaluation metric used is the average AUC score, calculated over 20 repetitions. In these simulations, the proportion of rare variants is set to zero.

**chr6:192074807-21684054**	**0%**	**25%**	**50%**	**75%**	**100%**
Epi-PRS	**0.7115 ± 0.0176**	0.7160 ± 0.0184	**0.7290 ± 0.0160**	0.7555 ± 0.0193	**0.7783 ± 0.0217**
Epi-PRS (blood features only)	0.6861 ± 0.0191	0.7070 ± 0.0199	0.7292 ± 0.0196	**0.7735 ± 0.0224**	0.7691 ± 0.0196
PRS-CS (without additional GWAS)	0.6649 ± 0.0211	0.6707 ± 0.0223	0.6625 ± 0.0235	0.6716 ± 0.0217	0.6724 ± 0.0199
LDPred2 (without additional GWAS)	0.6603 ± 0.0174	0.6801 ± 0.0202	0.6816 ± 0.0184	0.6794 ± 0.0180	0.6709 ± 0.0196
Genotype-PCA-GBRT	0.7044 ± 0.0201	**0.7169 ± 0.0203**	0.7170 ± 0.0159	0.7150 ± 0.0231	0.7178 ± 0.0129
Genotype-GBRT	0.7035 ± 0.0231	0.7109 ± 0.0160	0.7238 ± 0.0189	0.7066 ± 0.0135	0.7157 ± 0.0219

Table S4. Simulated Results with Varying Proportions of Epigenetic Effects. This table presents the simulated results for the genomic region chr6:31571218-32682664, highlighting the impact of different proportions of epigenetic effects on prediction performance. The evaluation metric used is the average AUC score, calculated over 20 repetitions. In these simulations, the proportion of rare variants is set to zero.

**chr6:31571218-32682664**	**0%**	**25%**	**50%**	**75%**	**100%**
Epi-PRS	0.7357 ± 0.0180	**0.7553 ± 0.0216**	**0.7656 ± 0.0149**	**0.7785 ± 0.0183**	0.7817 ± 0.0183
Epi-PRS (blood features only)	0.7344 ± 0.0182	0.7432 ± 0.0192	0.7619 ± 0.0174	0.7776 ± 0.0246	**0.7834 ± 0.0208**
PRS-CS (without additional GWAS)	0.6889 ± 0.0180	0.6842 ± 0.0167	0.6796 ± 0.0149	0.6779 ± 0.0170	0.6782 ± 0.0159
LDPred2 (without additional GWAS)	0.6736 ± 0.0192	0.6813 ± 0.0142	0.6813 ± 0.0169	0.6831 ± 0.0184	0.6804 ± 0.0245
Genotype-PCA-GBRT	0.7234 ± 0.0205	0.7386 ± 0.0202	0.7325 ± 0.0187	0.7494 ± 0.0220	0.7403 ± 0.0195
Genotype-GBRT	**0.7457 ± 0.0187**	0.7413 ± 0.0178	0.7330 ± 0.0198	0.7358 ± 0.0224	0.7447 ± 0.0240

Table S5. Simulated Results with Varying Proportions of Rare Variants. This table presents the simulated results for the genomic region chr6:192074807-21684054, highlighting the impact of different proportions of rare variants on prediction performance. When the proportion of rare variants increases to 100%, traditional PRS methods fail to predict effectively as they only consider common variants. The evaluation metric used is the average AUC score, calculated over 20 repetitions. In these simulations, the proportion of epigenetic effect is set to zero.

**chr6:192074807-21684054**	**0%**	**25%**	**50%**	**75%**	**100%**
Epi-PRS	**0.7486 ± 0.0154**	**0.7477 ± 0.0192**	**0.7522 ± 0.0192**	0.7362 ± 0.0166	**0.7510 ± 0.0246**
PRS-CS (without additional GWAS)	0.6169 ± 0.0183	0.6208 ± 0.0219	0.5668 ± 0.0195	0.5253 ± 0.0210	NA
LDPred2 (without additional GWAS)	0.6137 ± 0.0166	0.5945 ± 0.0189	0.5506 ± 0.0177	0.5215 ± 0.0232	NA
Genotype-PCA-GBRT	0.7410 ± 0.0191	0.7454 ± 0.0198	0.7433 ± 0.0263	**0.7406 ± 0.0179**	0.7450 ± 0.0170
Genotype-GBRT	0.7422 ± 0.0193	0.7423 ± 0.0177	0.7382 ± 0.0180	0.7368 ± 0.0222	0.7444 ± 0.0184

Table S6. Simulated Results with Varying Proportions of Rare Variants. This table presents the simulated results for the genomic region chr6:31571218-32682664, highlighting the impact of different proportions of rare variants on prediction performance. When the proportion of rare variants increases to 100%, traditional PRS methods fail to predict effectively as they only consider common variants. The evaluation metric used is the average AUC score, calculated over 20 repetitions. In these simulations, the proportion of epigenetic effect is set to zero.

**chr6:31571218-32682664**	**0%**	**25%**	**50%**	**75%**	**100%**
Epi-PRS	0.7420 ± 0.0196	**0.7650 ± 0.0152**	**0.7506 ± 0.0171**	0.7518 ± 0.0218	**0.7594 ± 0.0171**
PRS-CS (without additional GWAS)	0.6244 ± 0.0174	0.6196 ± 0.0233	0.5697 ± 0.0157	0.5557 ± 0.0135	NA
LDPred2 (without additional GWAS)	0.6386 ± 0.0247	0.5993 ± 0.0205	0.5598 ± 0.0221	0.5467 ± 0.0148	NA
Genotype-PCA-GBRT	0.7263 ± 0.0209	0.7538 ± 0.0171	0.7385 ± 0.0177	0.7501 ± 0.0199	0.7505 ± 0.0209
Genotype-GBRT	**0.7556 ± 0.0285**	0.7646 ± 0.0232	0.7351 ± 0.0176	**0.7596 ± 0.0203**	0.7516 ± 0.0181

Table S7. The performance of Epi-PRS and baseline methods on polygenic prediction of breast cancer based on 3 LD block without significant variants.

LD block	PRS-CS	LDPred2	Genotype-GBRT	Epi-PRS
chr4:5502388-6773043	0.4978	0.5048	0.5069	0.5055
chr8:19492840-20060856	0.5017	0.5102	0.4917	0.4976
chr19:43862455-44744108	0.5041	0.5018	0.5123	0.5048

Table S8. The performance of Epi-PRS for breast cancer was evaluated by varying the number of PCs in two LD blocks. The results indicate that using five PCs captures most of the variance while maintaining computational efficiency.

**Number of PC**	**2**	**3**	**5**	**10**
chr16:52035823-5338257	0.5808	0.5892	**0.5917**	0.5901
chr22:27834752-29651799	0.5392	0.5444	0.5497	**0.5499**
